# Incidence and predictors of fragility fracture in postmenopausal rheumatoid arthritis patients receiving oral bisphosphonates: a longitudinal observational study

**DOI:** 10.1186/s41927-021-00243-x

**Published:** 2022-02-28

**Authors:** Yuji Kishimoto, Yoshihiro Kato, Manami Uemura, Koji Kuranobu

**Affiliations:** 1 Department of Rheumatology, Tottori Red Cross Hospital, 117 Shotoku-cho, Tottori-shi, Tottori 680-8517 Japan; 2 Department of Orthopedic Surgery, Tottori Red Cross Hospital, Tottori, 680-8517 Japan; 3 Department of Rehabilitation, Tottori Red Cross Hospital, Tottori, 680-8517 Japan

**Keywords:** Bisphosphonate, Fracture, Osteoporosis, Rheumatoid arthritis

## Abstract

**Background:**

Although many studies have reported the predictors of fractures in patients with rheumatoid arthritis (RA) who are not receiving anti-osteoporotic treatments or who are receiving unspecified treatments, studies focusing on the predictors of fracture in patients with RA who are currently being treated with oral bisphosphonates (BP) are quite scarce. This study aims to investigate the incidence and predictors of fragility fracture in postmenopausal patients with RA receiving oral BP.

**Methods:**

This retrospective longitudinal observational study comprised 98 postmenopausal RA patients receiving oral BP for a minimum of 6 months between April 2015 and December 2020. The cumulative incidence of fragility fractures including vertebral and nonvertebral fractures was investigated using the Kaplan–Meier method. Cox proportional hazards analysis was used to analyze baseline predictors of future fragility fractures. To determine a cutoff value of continuous predictors, the receiver-operating characteristic curve was applied.

**Results:**

Twenty patients developed fractures during the study period, with a cumulative incidence of 6.1% at 12 months, 10.5% at a median follow-up of 28 months, and 14.4% at 36 months. Multivariable Cox hazards analysis showed a history of prior vertebral fracture (hazard ratio [HR] 6.26, 95% confidence interval [CI] 1.99‒19.68, *P* = 0.001) and dose of methotrexate (HR 0.87, 95% CI 0.76‒0.99, *P* = 0.041) to be independent predictors. The cutoff value for methotrexate dose was 4 mg/week.

**Conclusions:**

We found a cumulative incidence of any fractures of 10.5% at 28 months in patients with RA currently being treated with oral BP. A history of prior vertebral fractures and methotrexate dose were positive and negative predictors for fractures, respectively. Practitioners should consider selecting another anti-osteoporotic drug in patients with RA who remain at risk despite receiving oral BP.

## Background

Rheumatoid arthritis (RA) is a chronic inflammatory disease that is frequently associated with localized and generalized osteoporosis. Previous studies have shown that the incidence of fractures in patients with RA is high [1–3], and although striking improvements have been made in the disease control of RA in recent years, the incidence has not decreased [4]. Osteoporotic fractures are associated with reduced functional status in RA patients [5]. Therefore, in conjunction with tight control of disease activity using disease-modifying antirheumatic drugs (DMARDs), the continuing of anti-osteoporotic treatment is mandatory [6].

Currently, there are various anti-osteoporotic drugs on the market, including oral bisphosphonates (BP), intravenous zoledronic acid, denosumab, teriparatide, and romosozumab in Japan. Among these drugs, oral BP, because of their efficacy and safety combined with low cost, are most commonly used [7] as first-line drugs [8]. Previous studies have concluded that the use of oral BP significantly increased bone mineral density (BMD) [9] and had a protective effect against future fractures in patients with RA [2].

In contrast, several studies have reported that switching from BP to another anti-osteoporotic drug, including denosumab [10, 11] or teriparatide [11, 12], resulted in significant increases in BMD in patients with RA. Furthermore, in patients with glucocorticoid-induced osteoporosis (GIO), incident vertebral fractures were significantly decreased in patients treated with teriparatide as compared with those treated with alendronate [13]. These results suggest that the substitution of a recent anti-osteoporotic drug such as denosumab and teriparatide for oral BP might result in better improvement in BMD and subsequent decreases in future fractures in patients with RA. Therefore, understanding the predictors of fractures is useful when deciding between anti-osteoporotic drugs, to allow identification of patients at risk of future fracture despite receiving oral BP. However, although many studies have reported the predictors of fractures in patients with RA who are not receiving anti-osteoporotic treatments [14, 15] or who are receiving unspecified treatments [2, 16–18], studies focusing on the predictors of fracture in patients with RA who are currently being treated with oral BP are quite scarce.

The present study aimed to investigate the cumulative incidence of an endpoint defined as the development of fragility fractures in postmenopausal patients with RA who received oral BP for a minimum of 6 months. Furthermore, we also investigated baseline predictors associated with the development of future fragility fractures.

## Methods

### Study design and patients

This was a retrospective longitudinal observational study. All procedures were conducted in accordance with the Declaration of Helsinki and approved by the institutional review board of our institution.

The study population consisted of consecutive patients with RA treated in our hospital between April 2015 and December 2020. All eligible patients fulfilled the inclusion criteria as follows: postmenopausal women aged ≥ 50 years with RA who met the 2010 American College of Rheumatology/European League Against Rheumatism criteria [19], patients who met our indication of pharmacologic treatment for osteoporosis in patients with RA as described later in this paper, and patients who were newly prescribed oral BP. The exclusion criteria included patients who had previously received a selective estrogen receptor modulator, teriparatide, denosumab, and/or romosozumab; patients who had not continued their prescription of oral BP for more than 6 months; and patients who developed fractures within 6 months from the initiation of oral BP.

### Evaluation and indication of pharmacologic treatment for osteoporosis

In the osteoporosis evaluation, participants were asked about their history of fractures and underwent thoracic and lumbar spine radiologic examination as well as dual X-ray absorptiometry (DXA) at the hip and spine. We initiated pharmacologic treatment with oral BP for osteoporosis in patients with RA who met any one of the following criteria, in this order: (1) history of a fragility fracture at the vertebra and/or proximal femur, (2) T-score <  − 2.5 of BMD at the lumbar spine and/or total hip as measured by DXA, and (3) fulfilling the Japanese Society of Osteoporosis criteria for the diagnosis of GIO [20]. We defined fragility fractures as a bone fracture resulting from a fall from standing height or lower or without any trauma. We performed DXA scans using a Hologic QDR2000 scanner (Hologic Inc., Waltham, MA, USA).

### Oral BP prescription

We prescribed either alendronate, risedronate, or minodronate as oral BP. The selection of oral BP was based on the discretion of the attending rheumatologist. We took the following measures to ensure adherence to oral BP. First, we preferentially selected monthly or weekly oral BP over daily oral BP [21, 22]. Second, at the initiation of oral BP, patients received an individual face-to-face educational meeting with a Certified Nurse by Japan Rheumatism Foundation (M.U.) and received a booklet that contained general information regarding osteoporosis. Third, patients provided a self-reported recall of adherence at each visit.

### Diagnosis of fragility fractures

After the patient began taking oral BP agents, we monitored for the development of subsequent fragility fractures. We diagnosed symptomatic fragility fractures when the patient experienced an acute increase in pain as a result of a fall from standing height or lower with radiologic evidence of fractures. Furthermore, to evaluate asymptomatic subsequent vertebral fractures, we obtained thoracic and lumbar spine radiographs every 6–12 months after the initiation of oral BP until the final follow-up. We defined asymptomatic vertebral fractures as a new fracture with a decrease of ≥ 20% in any vertebral height from baseline [23].

### Potential predictors

In the present study, we determined potential predictors of fragility fractures based on the existing knowledge of predictors of fractures in patients with RA (such as age [2, 16, 17], body mass index [14, 17], disease duration [14], history of prior fracture [2, 16], disease activity [15, 16], functional impairment [16, 17], rheumatoid factor [15], daily oral glucocorticoid [16, 18], and BMD of the spine and hip [14, 15]) at baseline. Furthermore, we also included potential predictors of osteoporosis in patients with RA (such as anti-citrullinated peptide antibody [24], methotrexate [25, 26], and biological DMARDs [27]) as well as thorough discussions with attending physicians specializing in rheumatology (such as vitamin D supplementation and type of oral BP).

To evaluate the patients’ history of prior vertebral fracture, we obtained thoracic and lumbar spine radiographs at baseline. We did not include history of prior nonvertebral fracture, as only three patients confirmed this history. Disease activity and functional impairment were estimated using the Simplified Disease Activity Index (SDAI) and the Health Assessment Questionnaire Disability Index (HAQ-DI), respectively. Rheumatoid factor and anti-citrullinated peptide antibody were evaluated based on both their positivity and titers. We evaluated oral glucocorticoid and methotrexate based on whether the patient was taking these as well as the patient’s daily and weekly dose, respectively. We assessed the use of biological DMARDs and vitamin D supplementation by whether the patient was taking these at baseline.

### Statistical analyses

To estimate the cumulative incidence of any fragility fracture after the prescription of oral BP, we performed Kaplan–Meier analyses, and the comparison was performed by log-rank test, with a *P* value of < 0.05 considered statistically significant. Observations were right-censored at the last date at which thoracic and lumbar spine radiography was conducted, if fractures had not developed clinically or radiologically. We presented the cumulative incidence as the percentage and 95% confidence intervals (CIs).

We used the Cox proportional hazards model to estimate the hazard ratios (HRs) of predictors related to fragility fractures by univariate and multivariate analyses. Because the calculation of large numbers of variables on a limited number of patients might lead to overfitting, we selected variables for the identification of a useful, restricted subset of variables for predictors related to fragility fractures. First, we applied the univariate analyses, and if a variable had a *P* value of < 0.05 in the univariate analyses, we used it in the multivariate analysis. In the multivariate analysis, all variables with a *P* value of < 0.05 were considered statistically significant and as independent predictors. For a continuous variable detected as an independent predictor, we used the receiver-operating characteristic (ROC) curve to determine a cutoff value for predicting the development of any fragility fracture. The point closest to the upper left-hand corner of the graph was chosen as the cutoff value.

We conducted statistical analyses using R, version 3.4.1 (The R Foundation for Statistical Computing, Vienna, Austria).

## Results

### Patient characteristics

A total of 105 postmenopausal women aged ≥ 50 years with RA fulfilled the inclusion criteria of this study. Of these 105 patients, 7 were excluded: 4 had previously received selective estrogen receptor modulator and 3 were prescribed oral BP for less than 6 months. Finally, we included 98 patients in the present study. Of these 98 patients, 28 were prescribed oral BP for a history of fragility fracture, 61 were for a T-score <  − 2.5 of BMD, and 9 were for fulfilling the diagnosis of GIO in terms of the indication of pharmacologic treatment for osteoporosis.

Table 1 displays the characteristics of the 98 patients, including demographic and clinical variables and medication use at the time of oral BP initiation (d). The median (interquartile range) patient age at baseline was 69 (65–74) years. The median duration of disease was 80 (8–209) months, the SDAI was 6.80 (3.83–12.00), and the HAQ-DI was 0.50 (0.13–1.13). Twenty-seven (27.6%) patients had a history of prior vertebral fractures. Methotrexate was concomitantly administered in 79.6% of patients with a median dose of 6 mg/week, whereas oral glucocorticoid was concomitantly administered in 50.0% of the patients with a median dose of 0.2 mg/day. The methotrexate dose in the present study was comparable with that used in recent multicenter observational studies performed in Japan [28, 29]. Seventy-two (73.5%) patients were prescribed monthly oral BP, and 26 (26.5%) were prescribed weekly oral BP. All patients who were prescribed alendronate received one dose weekly (35 mg), and those prescribed minodronate received one dose monthly (50 mg). Risedronate was administered monthly (75 mg) to 12 patients and weekly (17.5 mg) to 12 patients. The median follow-up period, defined as the length of BP treatment, was 28 (11–45) months. The median length of BP treatment in patients with and without fractures was 35 (16–49) months and 28 (11–44) months, respectively; no statistically significant difference was observed between the two groups (*P* = 0.282; Mann–Whitney *U* test).

**Table 1:** Patient characteristics at baseline

Parameter	Total (n = 98)	Fractured (n = 20)	Non-fractured (n = 78)
Age (years old)	69 (65–74)	71 (67–75)	69 (65–74)
BMI (kg/m^2^)	20.4 (18.7–22.9)	20.1 (17.8–21.2)	20.5 (19.0–23.4)
Disease duration (months)	80 (8–209)	180 (56–270)	63 (6–162)
History of vertebral fracture, n (%)	27 (27.6)	14 (70.0)	13 (16.7)
SDAI	(3.83–12.00)	7.24 (4.70–11.87)	6.73 (3.72–12.00)
HAQ-DI	0.50 (0.13–1.13)	1.00 (0.50–1.63)	0.44 (0.13–1.00)
Rheumatoid factor positive, n (%)	72 (73.5)	16 (80.0)	56 (71.8)
Rheumatoid factor (IU/mL)	45 (14–89)	41 (23–80)	45 (14–89)
ACPA positive, n (%)	75 (76.5)	16 (80.0)	59 (75.6)
ACPA (U/mL)	25 (5–250)	33 (8–366)	35 (3–234)
CRP (mg/dL)	0.18 (0.04–0.67)	0.09 (0.01–0.83)	0.19 (0.05–0.58)
Concomitant oral glucocorticoid, n (%)	49 (50.0)	13 (65.0)	36 (46.1)
Dose of oral glucocorticoid (mg/day)	0.2 (0–3.8)	2.5 (0–3.0)	0 (0–5.0)
Dose of oral glucocorticoid^a^ (mg/day)	4.0 (2.5–5.0)	3.0 (2.5–3.5)	5.0 (2.5–7.5)
Concomitant methotrexate, n (%)	78 (79.6)	16 (80.0)	62 (79.5)
Dose of methotrexate (mg/week)	6.0 (2.0–8.0)	4.0 (2.0–8.0)	8.0 (4.0–8.0)
Dose of methotrexate^a^ (mg/week)	8.0 (6.0–10.0)	6.0 (4.0–8.5)	8.0 (6.0–10.0)
Concomitant bDMARDs, n (%)	33 (33.7)	9 (45.0)	24 (30.8)
Lumbar BMD (g/cm^2^)	0.73 (0.66–0.82)	0.73 (0.63–0.83)	0.73 (0.66–0.81)
Total hip BMD (g/cm^2^)	0.62 (0.56–0.68)	0.56 (0.50–0.65)	0.62 (0.58–0.68)
Vitamin D supplementation, n (%)	27 (27.6)	3 (15.0)	24 (30.8)
*Type of oral BP, n (%)*			
Alendronate	14 (14.3)	5 (25.0)	9 (11.5)
Risedronate	24 (24.5)	6 (30.0)	18 (23.1)
Minodronate	60 (61.2)	9 (45.0)	51 (65.4)
Length of BP treatment (months)	28 (11–45)	35 (16–49)	28 (11–44)

Continuous variables were presented as the median with interquartile range, and categorical variables were presented as numbers with percentages

BMI, Body mass index; SDAI, Simplified Disease Activity Index; HAQ-DI, Health Assessment Questionnaire Disability Index; ACPA, anticyclic citrullinated protein antibodies; CRP, C-reactive protein; bDMARDs, biological disease-modifying antirheumatic drugs; BMD, bone mineral density; BP, bisphosphonates

^a^Median (interquartile range) among patients receiving drugs

### Cumulative incidence of fragility fracture

**Fig. 1:**
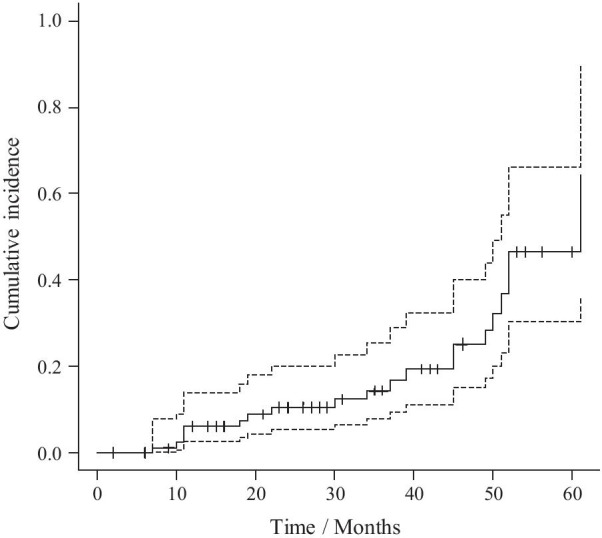
Cumulative incidence curve with 95% confidence intervals for the endpoint defined as the development of any fragility fractures

During the study period, 20 of 98 patients developed fragility fractures. Fracture regions included the vertebrae in nine patients, the distal forearm in four patients, the proximal humerus in three patients, the femoral neck in two patients, and pelvis and rib in one patient each. Figure 1 presents the cumulative incidence of fragility fractures, which was 6.1% (95% CI 0.8–11.1) at 12 months, 10.5% (95% CI 3.3–17.2) at a median follow-up of 28 months, and 16.8% (95% CI 6.8–25.8) at 36 months after initiation of oral BP.

### Predictors for fragility fracture

Table 2 shows the results of the univariate and multivariable Cox proportional hazards analyses for fragility fractures. The univariate Cox proportional hazards analysis identified a history of prior vertebral fracture (HR 5.67, 95% CI 2.17–14.79, *P* < 0.001), HAQ-DI (HR 1.91, 95% CI 1.10–3.31, *P* = 0.020), concomitant oral glucocorticoid use (HR 2.52, 95% CI 1.00–6.37, *P* = 0.049), and dose of methotrexate (HR 0.82, 95% CI 0.73–0.92, *P* < 0.001) as significant factors associated with the development of fragility fractures. Using the above-mentioned four significant factors from the univariate analysis, the multivariable Cox proportional hazards analysis showed that prior vertebral fracture was an independent positive predictor (HR 6.26, 95% CI 1.99–19.68, *P* = 0.001) and that methotrexate dose was a significant negative predictor (HR 0.87, 95% CI 0.76–0.99, *P* = 0.041) for fragility fractures in postmenopausal patients with RA receiving oral BP.

**Table 2:** Results of univariate and multivariate Cox proportional hazards analyses for fragility fractures in postmenopausal patients with rheumatoid arthritis receiving oral bisphosphonates

Variables	Univariable regression			Multivariable regression		
	HR	95% CI	*P* value	HR	95% CI	*P* value
Age	1.01	0.95–1.07	.661			
BMI	0.96	0.82–1.14	.716			
Disease duration	1.00	0.99–1.00	.117			
History of vertebral fracture	5.67	2.17–14.79	< .001	6.26	1.99–19.68	.001
SDAI	0.98	0.92–1.04	.519			
HAQ-DI	1.91	1.10–3.31	.020	1.34	0.74–2.45	.325
Rheumatoid factor positive	2.51	0.81–7.07	.107			
Rheumatoid factor titer	1.00	0.99–1.00	.900			
ACPA positive	1.76	0.57–5.40	.323			
ACPA titer	1.00	0.99–1.00	.398			
CRP	1.12	0.71–1.75	.621			
Concomitant oral glucocorticoid	2.52	1.00–6.37	.049	2.02	0.70–5.81	.190
Dose of oral glucocorticoid	1.10	0.93–1.29	.255			
Concomitant methotrexate	0.34	0.11–1.05	.061			
Dose of methotrexate	0.82	0.73–0.92	< .001	0.87	0.76–0.99	.041
Concomitant bDMARDs	1.61	0.65–3.98	.297			
Lumbar BMD	0.48	0.00–28.29	.724			
Total hip BMD	0.01	0.00–3.19	.104			
Vitamin D supplementation	0.56	0.16–1.96	.371			
Minodronate	0.56	0.18–1.64	.303			
Risedronate	0.70	0.21–2.36	.575			

HR, Hazard ratio; 95% CI, 95% confidence interval; BMI, body mass index; SDAI, Simplified Disease Activity Index; HAQ-DI, Health Assessment Questionnaire Disability Index; ACPA, anticyclic citrullinated protein antibodies; CRP, C-reactive protein; bDMARDs, biological disease-modifying antirheumatic drugs; BMD, bone mineral density; BP, bisphosphonates

**Fig. 2:**
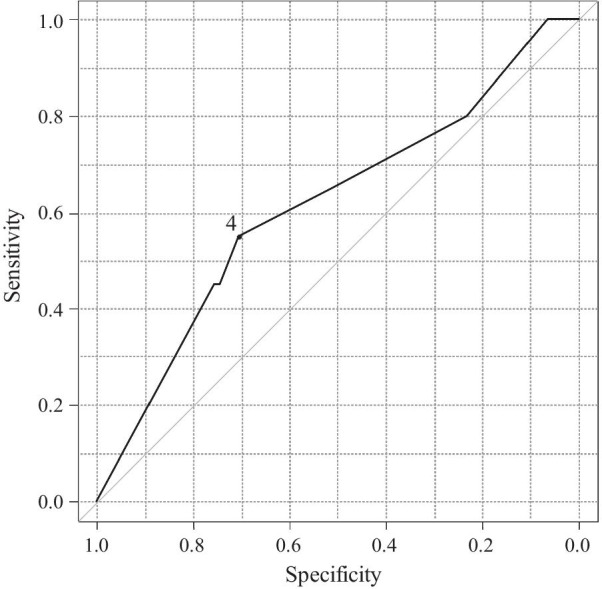
Receiver-operating curve analysis for evaluating the value of the baseline dose of methotrexate for predicting the development of future fragility fracture. The area under the curve was 0.61 (95% CI 0.47–0.75). A cutoff value of 4 mg/week was determined by the point closest to the upper left-hand corner of the graph with a sensitivity and specificity of 55.0% and 70.5%, respectively

To evaluate the cutoff value of the continuous predictor, the dose of methotrexate, we performed ROC analysis (Fig. 2). The area under the curve was 0.61 (95% CI 0.47–0.75). A cutoff value of 4 mg/week was determined by the point closest to the upper left-hand corner of the graph with a sensitivity and specificity of 55.0% and 70.5%, respectively.

### Subgroup analysis between patients with or without predictors for fragility fractures

**Fig. 3:**
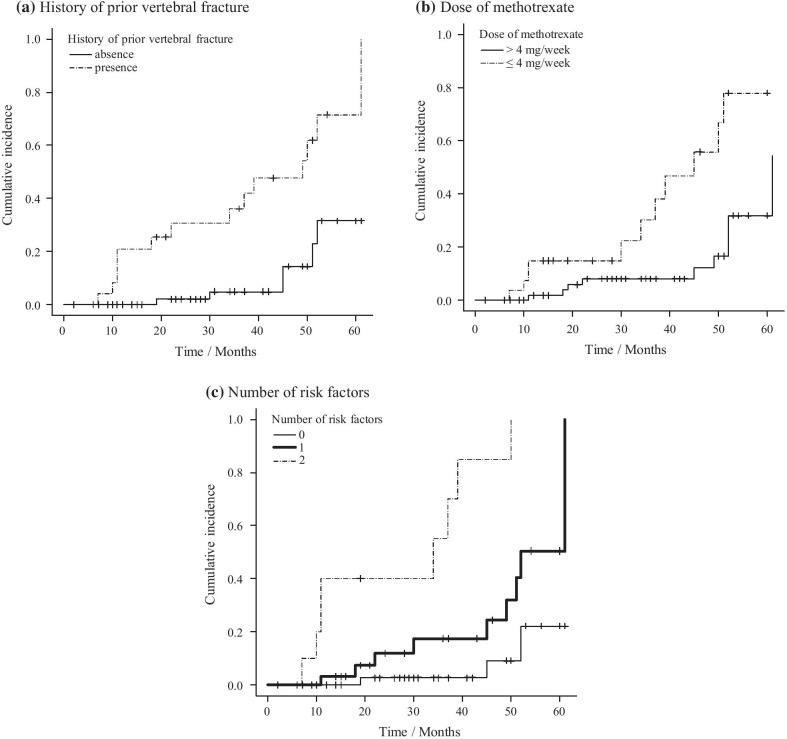
Cumulative incidence of fragility fractures in different subgroups. **a** History of prior vertebral fracture. **b** Dose of methotrexate. **c** Number of risk factors. Figure 3 showed the cumulative incidence of fragility fractures with or without predictors. The endpoint was defined as the development of any fragility fractures. Subgroups were divided according to the following definitions; **a** presence or absence of a history of fractures, **b** > 4 or ≤ 4 mg/week of dose of methotrexate, and **c** number of risk factors (0–2). Risk factors included a history of prior vertebral fracture and ≤ 4 mg/week dose of methotrexate

Based on the assessment of predictors for fragility fractures, we performed subgroup analysis for the cumulative incidence of any fragility fracture. The cumulative incidence at 28 months in patients who had an absence (n = 71) and presence (n = 27) of a history of prior vertebral fracture was 2.1% (95% CI 0–11.4) and 30.8% (95% CI 8.6–47.6), respectively (*P* < 0.001; Fig. 3a). At 28 months, the cumulative incidence in patients prescribed > 4 mg/week (n = 64) and ≤ 4 mg/week (n = 34) dose of methotrexate was 8.0% (95% CI 0.1–15.2) and 14.8% (95% CI 0.3–27.2), respectively (*P* < 0.001; Fig. 3b).

Considering a history of prior vertebral fracture and ≤ 4 mg/week dose of methotrexate to be risk factors for future fragility fracture, patients were categorized into three groups based on the number of risk factors they had: 0 (n = 49), 1 (n = 37), and 2 (n = 12). The cumulative incidence of any fragility fracture at 28 months in patients with 0, 1, and 2 risk factors was 2.7% (95% CI 0–8.0), 12.0% (95% CI 0–24.1), and 40.0% (95% CI 0.4–63.8), respectively (*P* < 0.001; Fig. 3c).

## Discussion

In this study, we investigated the incidence and predictors of fragility fractures in postmenopausal patients with RA who were prescribed oral BP. The cumulative incidence of fragility fracture was 10.5% at a median follow-up of 28 months. We found that a history of prior vertebral fracture and methotrexate dose were positive and negative predictors, respectively, of the development of future fragility fractures. The cutoff dose of methotrexate for future fragility fracture was 4 mg/week. The cumulative incidence of fragility fracture in the patients with both risk factors, a history of prior vertebral fracture and ≤ 4 mg/week dose of methotrexate, was significantly higher than the patients without risk factors.

There are only scarce studies investigating the mid- to long-term incidence of fractures in patients with RA receiving anti-osteoporotic medication (including oral BP). Kwon et al. reported a 3-year fracture incidence of 17.4% in Asian patients with RA aged 50 years or older from a nationwide claim database, with no mention of osteoporosis treatment [30]. Katayama et al. reported that 12.5% of patients older than 50 years old with RA who were prescribed alendronate developed vertebral or nonvertebral fracture during an average follow-up period of 32.8 months [31]. Taking into consideration our result of a 10.5% cumulative incidence of fracture at 28 months, we observed a certain degree of fracture prevention effect, however, some patients with RA are still at risk, even if they are receiving oral BP. Other new anti-osteoporotic drugs have recently become available, such as denosumab and teriparatide, which probably have better fracture prevention effects in patients with RA [10–12]. However, initiating treatment with these recent anti-osteoporotic drugs in all RA patients is very likely to be too costly; therefore, it is important to select patients at risk despite the prescription of oral BP.

In the present study, we identified a history of prior vertebral fracture as a positive predictor of fragility fractures. A systematic review on osteoporosis concluded that a history of prior fracture was an important risk factor for future fractures [32]. Furthermore, several previous studies found that in patients with RA, a history of prior fracture was a risk factor for incident fractures [2, 16, 33] with or without anti-osteoporotic medications. Our results suggest that in postmenopausal RA patients, a history of vertebral fracture is a strong predictor of fragility fractures, even among those who are taking oral BP medication.

In the present study, we found that the dose of methotrexate was a negative predictor and a dose of ≤ 4 mg/week dose was a risk factor for future fragility fractures. We suggest that there are two possible reasons why the outcome of methotrexate dose is a negative predictor. First, one of the reasons for restricting the dose of methotrexate is associated renal dysfunction that increases the incidence of any fracture risk [34]. Renal dysfunction in patients receiving no or low-dose methotrexate might affect the development of fragility fractures; however, we did not include an assessment of renal function in the present study. Second, previous studies reported that a combination of methotrexate and BP prevented systemic bone loss in an in vivo animal model of arthritis [35, 36] as well as in patients with RA [37]. There is a need for future clinical investigations to determine whether the combination of methotrexate and BP can decrease the development of fragility fractures.

The percentage of patients treated with bDMARDs appears to be higher in patients with (45.0%) than without fractures (30.8%); however, concomitant bDMARDs use was not significant in univariate Cox proportional hazards analysis. A recent systematic review concluded that bDMARDs had no effect on the fracture risk in RA patients [38], which is consistent with the present study.

The present study had several limitations. First, this was a retrospective longitudinal study limited by the use of data collected from patient charts. Although we included almost every factor that correlated with fragility fractures in RA patients in previous studies, we did not evaluate factors that might have affected the development of fragility fracture, such as vitamin D status, renal dysfunction, smoking, alcohol intake, or the Fracture Risk Assessment Tool score. In particular, vitamin D status was reported as a risk factor for fractures in Japanese postmenopausal women with RA [39]. Second, we did not include bone turnover markers determined to be predictive of future fractures in elderly women in previous studies [40, 41]. However, to the best of our knowledge, no study reports that bone turnover markers are useful for predicting fractures in patients with RA. Third, in the present study, we treated alendronate, risedronate, and minodronate identically to oral BP. Several previous studies reported that these medications had different efficacy in patients with RA [31, 42, 43]. Thus, the type of oral BP might affect the outcome. However, in the univariate Cox regression analysis, we did not find the type of oral BP to be a statistically significant factor. Fourth, we took several measures to ensure medication adherence, but no specific instrument was used to assess adherence to oral BP. Finally, this was a single-center study with a small population of patients. Thus, our results on the predictors for fragility fractures should be interpreted with caution. Larger, multicenter studies are warranted to obtain greater accuracy and generalizability of the study findings.

The present study investigated the incidence and predictors of fragility fractures in postmenopausal women with RA associated with osteoporosis who is receiving oral BP. We found a cumulative incidence of any fragility fractures of 10.5% at a median follow-up of 28 months. A history of prior vertebral fractures was a positive predictor, whereas the dose of methotrexate was a negative predictor of the development of fragility fractures. Practitioners should consider selecting another anti-osteoporotic drug in patients with RA who remain at risk despite receiving oral BP.

## Data Availability

The datasets during and/or analyzed during the current study available from the corresponding author on reasonable request.

## Abbreviations

BMI: Body mass index
BP: Bisphosphonates
CI: Confidence interval
DMARD: Disease-modifying antirheumatic drug
DXA: Dual X-ray absorptiometry
GIO: Glucocorticoid-induced osteoporosis
HAQ-DI: Health Assessment Questionnaire Disability Index
HR: Hazard ratio
RA: Rheumatoid arthritis
ROC: Receiver-operating characteristic
SDAI: Simplified disease activity index
